# Clinical and Ethical Approaches to Medical Futility in End-of-Life Care: A Scoping Review

**DOI:** 10.7759/cureus.110486

**Published:** 2026-06-08

**Authors:** Gustavo Monsalve-Morales, Jacobo Echeverri-Hoyos, Jaime A Echeverri Franco, Eduardo Tuta-Quintero

**Affiliations:** 1 Research Group UBUNTU and INNOVARE Research Seedbed, Universidad Nacional Abierta y a Distancia (UNAD), Bogotá, COL; 2 Medicine, Institución Universitaria Visión de las Américas, Pereira, COL; 3 Pulmonology, Clinica de Alta Tecnologia Oncologos del Occidente Maraya, Pereira, COL; 4 Epidemiology and Public Health, Universidad de La Sabana, Chía, COL

**Keywords:** clinical prognosis, end-of-life care, ethical decision-making, medical futility, therapeutic proportionality

## Abstract

Medical futility (MF) remains a complex and debated concept in clinical practice, particularly among patients with terminal illness or limited life expectancy, largely due to inconsistencies in its definition and variability in its application, which complicate clinical decision-making. A scoping review was conducted following the methodologies proposed by Arksey, Levac, the Joanna Briggs Institute, and the Preferred Reporting Items for Systematic Reviews and Meta-Analyses extension for Scoping Reviews (PRISMA-ScR) guidelines to analyze evidence on therapeutic futility in terminally ill adults and the clinical, ethical, and prognostic criteria influencing end-of-life decision-making. A total of 36 published studies were included. Regarding study design, retrospective observational studies predominated (12/36, 33.3%), followed by qualitative studies (9/36, 25.0%), cross-sectional studies (6/36, 16.7%), and, to a lesser extent, prospective or cohort studies (3/36, 8.3%), mixed-methods or case-control studies (2/36, 5.6%), and one case report (1/36, 2.8%). Most studies focused on patients (21/36, 58.3%), primarily in critical care settings or among individuals with advanced disease. A considerable proportion exclusively evaluated healthcare professionals (10/36, 27.8%). Regarding the clinical context, ICUs represented the predominant clinical setting, including general, oncological, surgical, neurological, and medical intensive care units. Other settings included non-critical hospital environments (5/36, 13.9%), oncology-specific settings (3/36, 8.3%), prehospital or emergency care settings (2/36, 5.6%), and additional areas, such as psychiatry, bioethics, or palliative care (3/36, 8.3%). The most frequently identified criterion for MF was the use of treatments without expected clinical benefit (10/36, 27.8%). Ethical and clinical dimensions were addressed across all included studies, with a predominance of multidimensional approaches integrating principles, such as autonomy, beneficence, and justice. Small sample sizes and single-center designs predominated, particularly among qualitative and retrospective studies. MF is a complex and multidimensional concept influenced by clinical, ethical, and sociocultural factors. The heterogeneity in its definition and application complicates the standardization of decision-making, particularly in settings such as ICUs.

## Introduction and background

Medical futility (MF) is generally defined as a medical intervention that fails to provide meaningful benefit to the patient because it does not achieve its intended clinical objective or improve quality of life [1,2]. However, the absence of a universally accepted definition has led to substantial variability in its interpretation and application in clinical practice [2,3]. Decision-making regarding potentially futile treatments remains one of the most challenging aspects of modern medicine [2]. In high-complexity settings, such as intensive care units (ICUs), oncology, and palliative care, these decisions frequently generate ethical tensions, interprofessional conflicts, and challenges within the physician-patient-family relationship [1-3]. Furthermore, perceptions and management of MF are strongly influenced by contextual, cultural, religious, legal, and subjective factors, contributing to considerable heterogeneity in clinical practice [3,4].

The evaluation of MF requires a comprehensive and multidisciplinary approach that integrates scientific evidence with patients' values, family preferences, and religious or cultural beliefs [3,5]. Decisions regarding treatment limitation, including do-not-resuscitate (DNR) orders and the withdrawal of life-sustaining therapies, are frequently made late in the course of illness and often occur with limited involvement of patients and their families [5,6]. Variability in decisions to withhold, withdraw, or redirect treatment may also be associated with unrealistic expectations regarding prognosis and therapeutic outcomes [5,6]. Consequently, shared decision-making models have emerged as a fundamental strategy for improving end-of-life care and reducing the use of non-beneficial interventions [6,7].

Despite increasing interest in MF, important gaps persist in the literature regarding the integration of clinical, ethical, and sociocultural dimensions into healthcare decision-making, particularly in real-world clinical settings [8,9]. Most available studies focus on specific populations or contexts and employ heterogeneous methodologies and definitions of futility, limiting the comparability and generalizability of findings [7-9]. This scoping review aims to identify patterns, barriers, and facilitators related to the assessment and management of MF in clinical practice, thereby contributing to the development of strategies that support more ethical, consistent, and patient- and family-centered decision-making.

## Review

Methods

This scoping review was conducted to evaluate therapeutic proportionality and the futility of medical interventions in adult patients with terminal illness or limited life expectancy. The review followed the methodological framework proposed by Arksey et al. [10], subsequently refined by Levac et al. [11], in addition to the recommendations of the Joanna Briggs Institute for scoping reviews [12]. Reporting was performed in accordance with the Preferred Reporting Items for Systematic Reviews and Meta-Analyses extension for Scoping Reviews (PRISMA-ScR) guidelines [13].

Formulation of the Research Question

The research question was structured using the PICO (Population, Intervention, Comparison, and Outcome) framework. The population included adult patients (≥ 18 years) with terminal illness, irreversible conditions, or limited life expectancy. The intervention corresponded to the evaluation of potentially futile medical interventions, limitation of therapeutic effort, or therapeutic adequacy. The comparison included the absence of therapeutic limitation or conventional management. Finally, the outcomes focused on the clinical, ethical, and prognostic criteria used to define futility or therapeutic proportionality, as well as their impact on clinical decision-making at the end of life. Based on these elements, the guiding question was the following: What clinical, ethical, and prognostic criteria are used to evaluate the proportionality or futility of medical interventions in adult patients with terminal illness or limited life expectancy, and how do they influence healthcare decision-making?

Literature Search Strategy

The search strategy was designed to identify relevant studies in international biomedical databases, including PubMed/MEDLINE, Scopus, and Embase. Controlled vocabulary terms (MeSH and Emtree) and keywords related to "medical futility", "therapeutic proportionality", "limitation of therapeutic effort", "end-of-life care", "intensive care unit", and "decision-making" were used (Table 1).

**Table 1:** Search strategies

PubMed/MEDLINE - 1066 results
("medical futility"[Mesh] OR "medical futility" OR futility OR "futile care" OR "non-beneficial treatment" OR "nonbeneficial treatment" OR "potentially inappropriate treatment" OR "inappropriate care") AND ("end-of-life care" OR "end of life care" OR "terminal care"[Mesh] OR "palliative care"[Mesh] OR "terminally ill" OR "advanced disease") AND ("decision making"[Mesh] OR "decision making" OR "clinical decision making" OR "shared decision making") Filters: from 1000/1/1 - 2026/4/17
Scopus - 813 results
TITLE-ABS-KEY ( ( "medical futility" OR futility OR "futile care" OR "non-beneficial treatment" OR "potentially inappropriate treatment" OR "inappropriate care" ) AND ( "end-of-life care" OR "end of life care" OR "terminal care" OR "palliative care" OR "terminally ill" OR "advanced disease" ) AND ( "decision making" OR "clinical decision making" OR "shared decision making" ) )
Embase - 495 results
('medical futility'/exp OR 'medical futility' OR futility OR 'futile care' OR 'non beneficial treatment' OR 'potentially inappropriate treatment' OR 'inappropriate care') AND ('terminal care'/exp OR 'palliative care'/exp OR 'end of life care' OR 'terminally ill patient' OR 'advanced disease') AND ('decision making'/exp OR 'decision making' OR 'clinical decision making' OR 'shared decision making') AND [humans]/lim

Eligibility Criteria

Studies conducted in adult populations (≥ 18 years) with terminal illness, irreversible conditions, or limited life expectancy were included if they evaluated therapeutic futility, therapeutic proportionality, or the limitation/adequacy of therapeutic effort. Observational studies, clinical trials, qualitative research, systematic reviews, and bioethical analyses with a clinical basis were considered, provided that they were published in English or Spanish. Studies in pediatric populations, exclusively veterinary research, opinion articles without clinical support, editorials without structured analysis, protocols without results, and publications without full-text access were excluded.

Study Selection

Study selection was carried out in two phases. In the first phase, two independent reviewers screened titles and abstracts to identify potentially eligible studies using the Rayyan platform [14]. In the second phase, a full-text review of selected articles was performed to confirm final inclusion. Discrepancies between reviewers were resolved through discussion and consensus. The selection process was documented following the PRISMA flow diagram [13].

Data Extraction

Data extraction was performed using a previously designed standardized matrix to ensure consistency and comparability among included studies. Information collected from each article included author, year of publication, and country of origin; study design; characteristics of the clinical population and sample size; healthcare setting; criteria used to define futility or therapeutic proportionality; ethical or clinical dimensions addressed; main results; and limitations reported by the authors. This process allowed for systematic organization of the evidence and facilitated subsequent comparative analysis.

Synthesis and Analysis of Evidence

Given the heterogeneity of the included studies in terms of methodological design, populations, clinical settings, and definitions of futility, a narrative descriptive synthesis was conducted [15]. Studies were grouped according to the type of criteria evaluated (clinical, ethical, or prognostic), healthcare setting (such as ICU, oncology, or palliative care), and type of clinical decision (treatment limitation, withdrawal of life support, or do-not-resuscitate orders). This approach enabled the identification of common patterns, variability in clinical practice, and key factors influencing end-of-life decision-making, as well as highlighting the main knowledge gaps in the existing literature.

Results

**Figure 1:**
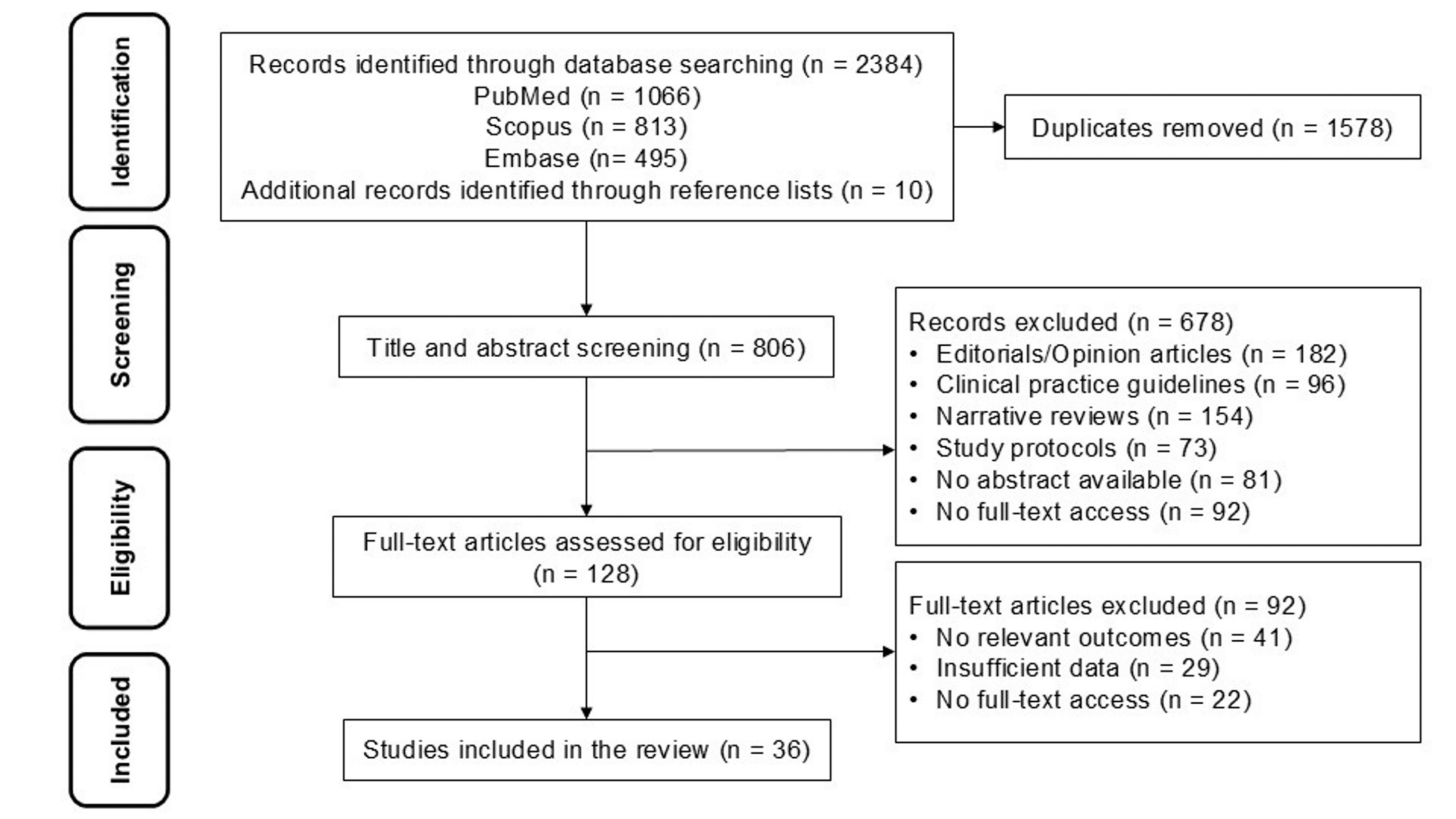
PRISMA-sc flowchart PRISMA-sc: Preferred Reporting Items for Systematic Reviews and Meta-Analyses extension for Scoping Reviews

A total of 36 published studies were included (Figure 1) [16-51]. Regarding geographical distribution, most studies were conducted in the United States (8/36, 22.2%) [17,22-24,26,27,36,51], followed by Australia (3/36, 8.3%) [21,28,30], France (3/36, 8.3%) [33,47,48], and South Korea (2/36, 5.6%) [18,32] (Table 2). Regarding study design, retrospective observational studies predominated (12/36, 33.3%) [18,20,23,26,28,32,34,39-41,46,50], followed by qualitative studies (9/36, 25.0%) [16,17,29-31,43,45,49,51], cross-sectional studies (6/36, 16.7%) [22,25,35,37,38,44], and, to a lesser extent, prospective or cohort studies (3/36, 8.3%) [21,27,33], mixed-methods or case-control studies (2/36, 5.6%) [36,42], and one case report (1/36, 2.8%) [24] (Table 3). Most studies focused on patients (21/36, 58.3%), primarily in critical care settings or among individuals with advanced disease [18,20,23,26,28,32-34,39-42,46,48,50]. A considerable proportion exclusively evaluated healthcare professionals (10/36, 27.8%) [17,22,25,30,31,37,38,43,44,49], while some studies incorporated mixed perspectives from patients, family members, and healthcare professionals (3/36, 8.3%) [16,36,45], and one study focused exclusively on surrogate family members (1/36, 2.8%) [51].

**Table 2:** Characteristics of the included studies and clinical settings AIDS, acquired immunodeficiency syndrome; BMI, body mass index; CPR, cardiopulmonary resuscitation; DFLST, decisions to forgo life-sustaining therapy; DNR, do-not-resuscitate; GCS, Glasgow Coma Scale; ICU, intensive care unit; LST, life-sustaining treatment; LTE, limitation of therapeutic effort; NBT, non-beneficial treatment; NFR, not-for-resuscitation; QOL, quality of life

Author/Country/Year	Clinical population	Futility criterion evaluated	Ethical/clinical dimension	Main result
Udeh et al. [16] - Nigeria - 2025	n=40 (10 patients, 10 family members, 20 professionals); patients aged 30-59; 5M/5F	Treatments without clinical benefit	Autonomy, beneficence, justice	Ethical conflicts and pressure for futile treatments
Piscitello et al. [17] - United States - 2025	n=16 professionals; age/sex not reported	Physiological futility	Clinical ethics	Variability in decisions
Kim et al. [18] - South Korea - 2023	n=227; mean age 66.2; 115M/112F	LST without benefit	Legal, family	Increased withdrawal of support after legislation
Martins et al. [19] - Portugal - 2019	n=120; elderly patients; caregivers mostly women	Acceptance/rejection of futile measures	Autonomy, religion	Unrealistic expectations about treatments
Nieder et al. [20] - Europe - 2011-2018	n=33; median age 70; 22M/11F	Low clinical utility	Proportionality	36% inappropriate decisions; low survival
Carter et al. [21] - Australia - 2019	n=831; mean age 72.3; 56% male	Non-beneficial treatment (NBT)	Ethical conflicts	12.4% NBT; strong association with conflicts
Chamberlin et al. [22] - United States - 2019	n=333 clinicians; age/sex not reported	Perception of futility	Burnout, moral distress	75.7% report futile care; association with burnout
Kamat et al. [23] - United States - 2012	n=53 oncology ICU patients; >70% life support	Futility and limitation of support	Communication, conflicts	↑ DNR (26→72%); high mortality (~77%)
Lopez et al. [24] - United States -	n=1 patient (female, 30 years; extreme BMI ~10-14)	Therapeutic futility due to complete treatment refractoriness	Autonomy, capacity, proportionality, treatment limits	Transition to palliative/hospice care due to lack of options
Palda et al. [25] - Canada - ~2005	n=255 professionals (141 nurses, 114 physicians) from ≈157 centers (64 complete)	Definition based on the Canadian Medical Association	Perception of futility, decision-making, ethical burden, institutional resources	High perceived frequency of futility (87-95% in the last year)
Rivera et al. [26] - United States - 2001	n=100 patients; mean age 73.5; 57% female	Treatment without benefit	Motivational factors	Unrealistic expectations
Curtis et al. [27] - United States - 2000	n=57 AIDS patients; age 39; 91% male	Low probability of benefit	Autonomy	61% accept limitation
Bloomer et al. [28] - Australia - 2010	n=70; mean age 69.3	Futility (60.7%)	End-of-life	High NFR and communication
Lonergan et al. [29] - United Kingdom - 2020	n=18 professionals	Dynamic futility	Clinical decision-making	Role of time
Willmott et al. [30] - Australia - 2016	n=96 physicians	Treatments without benefit	Multiple factors	Family pressure and curative culture
Beck et al. [31] - Germany -	n=28 ICU physicians; experience 1-34 years; mostly male	Decisions to limit life support (mechanical ventilation) based on prognosis and patient wishes	Ethical and legal confusion; difference between withdrawing vs withholding treatment	High confusion: many consider withdrawing ventilation illegal
Lee et al. [32]- South Korea - 2020	227 patients (80 in ICU)	Withdrawal vs withholding LST	Role of intensivist	↑ withdrawal with intensivists
Chauchard et al. [33] - France - 2026	73 ICU patients	Prognosis and quality of life	Legality and family	Withholding predominates, high satisfaction
Lee et al. [34] -Singapore - 2018	53 patients	Futility vs imminent death	Surgeon-intensivist conflict	71.7% conservative management
Druwé et al. [35] - International study (24 countries) - 2018	4018 CPR events reported by clinicians (physicians, nurses, paramedics)	Clinical perception of inappropriate CPR (perceived futility)	Clinical judgment, uncertainty, prognostic factors	8% CPR considered inappropriate; higher futility perception in patients ≥80 years
Zier et al. [36] - United States - 2009	50 surrogates / 31 patients	Medical prediction of futility	Family perception	64% distrust; continuation even with an extremely poor prognosis
Kadooka et al. [37] - Japan - 2012	401 physicians / 1134 general population	Clinical judgment vs social acceptance	Physician-society conflict	Laypeople more likely to treat; physicians prioritize prognosis and QOL
Pasieka et al. [38] - Poland - 2025	354 clinicians	Use of futility guidelines	Healthcare system	High theoretical acceptance, low implementation (35.3%)
Serenari et al. [39] - Multinational - 2025	788 patients	Early recurrence/early death	Clinical prediction	13.6% futile surgery; advanced tumor factors
Hariharan et al. [40] - Barbados - 2003	662 patients (30 futility)	Irreversible neurological status/non-response	Sociocultural factors	Continued treatment despite futility; ventilation never withdrawn
Jukić et al. [41] - Croatia - 2016	1567 patients	GCS <8 + intensive support	Real-world clinical practice	Persistence of support even without recovery expectation
Cruz et al. [42] - Brazil - 2015	276 patients (38 futility, 238 controls)	Life support in terminal patients without therapeutic options	Tumor type, palliative care involvement, age, communication	Palliative care ↓ futility; hematologic malignancies ↑ risk
Lo et al. [43] - Singapore - 2022	32 clinicians	Treatments perceived as non-beneficial	Family, cultural, clinical, institutional factors	Family as main driver; collectivist culture; variability among clinicians
Kalkan [44] - Turkey - 2018	376 physicians	CPR considered futile under specific conditions	Ethical/legal beliefs, experience, DNR perception	Only 47.7% would respect DNR; ethical/legal confusion; 57% see no dilemma in not resuscitating
Aizawa et al. [45] - Japan - 2013	19 participants	Treatments prolonging life without benefit and with suffering	Values, intentions, social consensus, family and medical perspectives	Futility depends on intention, suffering, and consensus
Becerra-Bolaños et al. [46] - Spain - 2024	80 patients with LTE (of 2055)	LTE: withholding, withdrawal, brain death	Age, comorbidity, severity, admission type, life support	5.1% LTE; withholding predominated (83.8%); mortality 93.7%; joint decisions
Ferrand et al. [47] - France -	816 patients	Withholding and withdrawal of life support	Age, chronic disease, prognosis, pain, quality of life, family	Withholding predominated (88%); decisions due to imminent death
Azoulay et al. [48] - France - 2012	7,899 patients (256 with birthdays during stay; 223 matched with 1,042 controls)	Prolongation of life support and delay in treatment limitation decisions (DFLST)	Clinical and ethical	ICU birthday patients had longer stays, higher treatment intensity, and delayed limitation decisions
Robertsen et al. [49] - Norway - 2019	18 physicians (neurosurgeons, intensivists, rehabilitation specialists)	Uncertainty about initiating, continuing, or withdrawing life support in severe brain injury	Ethical and clinical	Doubt is frequent; managed with “trial treatments,” time for reassessment, interdisciplinary discussion
Huang et al. [50] - Taiwan - 2010	873 deceased ICU patients (of 14,698 admissions)	DNR orders and treatment intensity changes	Clinical and ethical	High DNR rate (>65%); associated with lower treatment intensity
Parrish et al. [51] - United States - 2025	18 surrogate family members of critically ill patients	Potentially non-beneficial treatments in ICU	Ethical and clinical	Decision-making influenced by communication barriers

**Table 3:** Ethical and clinical dimensions and main findings of the studies on medical futility ICU, intensive care unit; HIV, human immunodeficiency virus

Author/Country/Year	Design	Clinical context
Udeh et al. [16] - Nigeria - 2025	Cross-sectional qualitative	Oncology and ICU
Piscitello et al. [17] - United States - 2025	Qualitative	ICU
Kim et al. [18] - South Korea - 2023	Retrospective	Neurosurgery
Martins et al. [19] - Portugal - 2019	Observational	Palliative care
Nieder et al. [20] - Europe - 2011-2018	Retrospective	Oncology
Carter et al. [21] - Australia - 2019	Multicenter cohort	Hospital
Chamberlin et al. [22] - United States - 2019	Cross-sectional	Academic hospital
Kamat et al. [23] - United States - 2012	Retrospective	Oncology ICU
Lopez et al. [24] - United States - 2010	Case report	Psychiatry/palliative care
Palda et al. [25] - Canada - ~2005	Cross-sectional	ICU in hospitals >200 beds
Rivera et al. [26] - United States - 2001	Retrospective	Tertiary hospital
Curtis et al. [27] - United States - 2000	Prospective	Advanced HIV
Bloomer et al. [28] - Australia - 2010	Retrospective	ICU
Lonergan et al. [29] - United Kingdom - 2020	Qualitative	ICU
Willmott et al. [30] - Australia - 2016	Qualitative	Hospital
Beck et al. [31] - Germany - 2008	Qualitative (interviews)	ICU
Lee et al. [32] - South Korea - 2020	Retrospective	ICU
Chauchard et al. [33] - France - 2026	Prospective	ICU France
Lee et al. [34] - Singapore - 2018	Retrospective	Surgical ICU
Druwé et al. [35] - International study (24 countries) - 2018	Cross-sectional	Emergency and prehospital services
Zier et al. [36] - United States - 2009	Mixed (observational)	ICU
Kadooka et al. [37] - Japan - 2012	Cross-sectional	End-of-life decisions
Pasieka et al. [38] -Poland - 2025	Cross-sectional	ICU
Serenari et al. [39] - Multinational -2025	Retrospective	Oncologic surgery
Hariharan et al. [40] - Barbados - 2003	Retrospective	Surgical ICU
Jukić et al. [41] - Croatia - 2016	Retrospective	ICU
Cruz et al. [42] - Brazil - 2015	Retrospective case-control	Oncology ICU
Lo et al. [43] - Singapore - 2022	Qualitative	Cardiology/ICU
Kalkan [44] - Turkey - 2018	Cross-sectional	Pulmonology/ICU
Aizawa et al. [45] - Japan - 2013	Qualitative	Bioethics/decision-making
Becerra-Bolaños et al. [46] - Spain - 2024	Retrospective	Post-surgical ICU
Ferrand et al. [47] - France - 2006	Cross-sectional	Prehospital care
Azoulay et al. [48] - France - 2012	Prospective	ICU
Robertsen et al. [49] - Norway - 2019	Qualitative	ICU
Huang et al. [50] - Taiwan - 2010	Retrospective	Surgical ICU
Parrish et al. [51] - United States - 2025	Qualitative	Medical ICU

Regarding the clinical context, ICUs represented the predominant clinical setting [16-18,23,25,28,29,31-35,38,40-44,46,48-51], including general, oncological, surgical, neurological, and medical ICUs. Other settings included non-critical hospital environments (5/36, 13.9%) [21,22,26,30,37], oncology-specific settings (3/36, 8.3%) [19,20,39], prehospital or emergency care settings (2/36, 5.6%) [35,47], and additional areas, such as psychiatry, bioethics, or palliative care (3/36, 8.3%) [24,27,45] (Table 3). The most frequently identified criterion for MF was the use of treatments without expected clinical benefit (10/36, 27.8%) [16,20,21,23,26,30,41-43,46], followed by futility based on poor physiological prognosis or low probability of survival (8/36, 22.2%) [17,18,34,35,40,41,48,50]. Other approaches included limitation of life-sustaining treatment through withholding or withdrawal decisions (6/36, 16.7%) [18,32,33,46-48], clinicians' subjective perceptions of futility (6/36, 16.7%) [22,25,35,36,37,44], and specific criteria related to cardiopulmonary resuscitation, surgical interventions, or legal frameworks (3/36, 8.3%) [35,39,44].

Ethical and clinical dimensions were addressed across all included studies, with a predominance of multidimensional approaches integrating principles such as autonomy, beneficence, and justice [16,24,27,45] (Table 2). Ethical conflicts and decision-making challenges were frequently reported (8/36, 22.2%) [16,21,23,29,31,34,43,51], as were the influence of family and sociocultural factors (6/36, 16.7%) [19,25,36,37,40,43] and legal or regulatory considerations (5/36, 13.9%) [18,31,33,44,48]. To a lesser extent, studies explored moral distress and burnout among healthcare professionals (3/36, 8.3%) [22,25,38], as well as issues related to therapeutic proportionality and clinical communication (4/36, 11.1%) [23,28,42,51].

Small sample sizes and single-center designs predominated, particularly among qualitative and retrospective studies [16,17,29,31,49], limiting the generalizability of the findings. Several studies based on surveys or clinicians' perceptions were subject to self-report and response bias [22,25,38,44], while retrospective designs were associated with potential selection, classification, and information biases [20,42,47,50]. Additionally, substantial heterogeneity was observed in the conceptualization and definition of MF, ranging from physiological criteria to subjective perceptions, sociocultural interpretations, and legal frameworks [17,35,37,45], complicating comparisons across studies and limiting the synthesis of evidence. A strong influence of cultural, legal, and institutional contexts was also identified [18,37,40,44], reducing the transferability of findings across different countries and healthcare systems.

Discussion

This scoping review shows that MF remains a complex and context-dependent concept, characterized by substantial variability in its definition and clinical application across healthcare settings and countries [16,17,35,37]. The absence of universally accepted criteria contributes to inconsistencies in decision-making and may generate conflicts among healthcare professionals, patients, and family members [16,36,43]. Across the included studies, futility was rarely determined solely by physiological prognosis or lack of therapeutic benefit; instead, ethical principles such as autonomy, beneficence, proportionality, and justice were consistently integrated into clinical decision-making [16,20,24,27]. These findings reinforce the idea that MF should be approached as a multidimensional construct requiring individualized and patient-centered assessment rather than exclusively biomedical evaluation [45,49].

The ICU emerged as the principal setting in which MF-related decisions occur, reflecting the high technological intensity and prognostic uncertainty characteristic of critically ill patients [17,28,33,48]. Several studies highlighted that uncertainty regarding prognosis, combined with variability in professional experience and ethical interpretation, may contribute to the continuation of potentially non-beneficial treatments and delayed limitation of life-sustaining therapies [23,29,49]. Communication barriers and unrealistic expectations among families were also recurrent factors associated with the persistence of futile interventions [19,26,36,51]. In parallel, healthcare professionals frequently reported moral distress, burnout, and ethical burden associated with the provision of care perceived as futile [22,25], emphasizing the need for structured communication strategies and shared decision-making models capable of aligning treatment goals with patient values and prognosis [21,36].

The findings also demonstrate that sociocultural, legal, and institutional contexts strongly influence perceptions and management of MF [18,31,37,40,44]. While some healthcare systems prioritize treatment continuation despite poor prognosis, others adopt more restrictive approaches focused on quality of life and proportionality of care. These differences highlight the absence of a universally standardized approach and support the need for ethical and regulatory frameworks adapted to local cultural and healthcare realities while preserving core bioethical principles [31,45]. Moreover, institutional factors such as the availability of palliative care services, ethical support, and futility guidelines may reduce variability in clinical practice and facilitate more consistent end-of-life decision-making [38,42].

Another relevant finding is the marked variability in how healthcare professionals define and operationalize MF. Several studies showed inconsistencies regarding the thresholds for withholding or withdrawing treatment, perceptions of inappropriate care, and interpretation of prognostic uncertainty [17,31,35,44,49]. This lack of standardization may contribute to unequal clinical practices, ethical conflicts, and prolonged use of non-beneficial interventions. The development of clearer conceptual and institutional frameworks could support more consistent and transparent decision-making processes [37,38].

The evidence suggests that improving the management of MF requires a multidimensional approach integrating clinical prognosis, ethical deliberation, communication skills, and sociocultural considerations. Strategies such as early palliative care integration, time-limited therapeutic trials, interdisciplinary discussions, and clearer institutional protocols may help reduce the use of disproportionate interventions and improve patient-centered care [24,29,31,42,49]. However, the predominance of observational and qualitative designs, together with substantial conceptual heterogeneity among studies, limits the comparability of findings. Future research using more robust and standardized methodological approaches is needed to strengthen the evidence base and support the development of clearer clinical and ethical frameworks for addressing MF [16,22,35,38].

Limitations and Strengths

This review has several limitations that should be considered when interpreting the findings. First, as a scoping review, its primary purpose was to map and characterize the breadth of the available literature on MF rather than to provide quantitative synthesis or formal comparative analysis, which inherently results in a more descriptive approach than that of systematic reviews or meta-analyses. Although the analytical component of the manuscript was strengthened through thematic integration and synthesis of findings, the exploratory nature of the review limited the possibility of performing statistical analyses or quantitative assessments of heterogeneity.

Additionally, although the search strategy included major biomedical databases such as PubMed/MEDLINE, Scopus, and Embase, grey literature was not included, which may have excluded relevant non-indexed evidence. The available studies also showed substantial methodological and conceptual heterogeneity, including differences in study design, clinical contexts, definitions of MF, and outcome measures. A considerable proportion of the included studies were retrospective or qualitative, many with single-center designs and relatively small sample sizes, limiting the generalizability and comparability of findings. Furthermore, several studies relied heavily on subjective perceptions of futility and lacked standardized operational criteria, making evidence integration more challenging.

Despite these limitations, this review provides a broad overview of the ethical, clinical, and sociocultural dimensions of MF across diverse healthcare settings. In addition, prior registration of the review protocol in the Open Science Framework (OSF) [52] strengthens the methodological transparency, reproducibility, and rigor of the study.

## Conclusions

MF is a complex and multidimensional concept, influenced by clinical, ethical, and sociocultural factors. Heterogeneity in its definition and application complicates the standardization of decision-making, particularly in settings such as the ICU. The findings of this review highlight the need to integrate clinical criteria with patient values, strengthen communication, and promote shared decision-making. The development of clearer conceptual frameworks and clinical tools is required to better guide clinical practice.

## References

[1] Bibas L, Peretz-Larochelle M, Adhikari NK (2019). Association of surrogate decision-making interventions for critically ill adults with patient, family, and resource use outcomes: a systematic review and meta-analysis. JAMA Netw Open.

[2] Bernat JL (2005). Medical futility: definition, determination, and disputes in critical care. Neurocrit Care.

[3] Bosslet GT, Pope TM, Rubenfeld GD (2015). An official ATS/AACN/ACCP/ESICM/SCCM policy statement: responding to requests for potentially inappropriate treatments in intensive care units. Am J Respir Crit Care Med.

[4] White DB, Curtis JR, Lo B, Luce JM (2006). Decisions to limit life-sustaining treatment for critically ill patients who lack both decision-making capacity and surrogate decision-makers. Crit Care Med.

[5] Javanmard-Emamghissi H, Moug SJ (2022). The virtual uncertainty of futility in emergency surgery. Br J Surg.

[6] Lambden JP, Chamberlin P, Kozlov E (2019). Association of perceived futile or potentially inappropriate care with burnout and thoughts of quitting among health-care providers. Am J Hosp Palliat Care.

[7] Torke AM, Sachs GA, Helft PR, Montz K, Hui SL, Slaven JE, Callahan CM (2014). Scope and outcomes of surrogate decision making among hospitalized older adults. JAMA Intern Med.

[8] Huynh TN, Kleerup EC, Raj PP, Wenger NS (2014). The opportunity cost of futile treatment in the ICU. Crit Care Med.

[9] Ely EW, Azoulay E, Sprung CL (2019). Eight things we would never do regarding end-of-life care in the ICU. Intensive Care Med.

[10] Arksey H, O’Malley L (2005). Scoping studies: towards a methodological framework. Int J Soc Res Methodol.

[11] Levac D, Colquhoun H, O'Brien KK (2010). Scoping studies: advancing the methodology. Implement Sci.

[12] Aromataris E, Munn Z (2026). JBI manual for evidence synthesis. JBI.

[13] Tricco AC, Lillie E, Zarin W (2018). PRISMA extension for scoping reviews (PRISMA-ScR): checklist and explanation. Ann Intern Med.

[14] Ouzzani M, Hammady H, Fedorowicz Z, Elmagarmid A (2016). Rayyan - a web and mobile app for systematic reviews. Syst Rev.

[15] Grudniewicz A, Nelson M, Kuluski K (2016). Treatment goal setting for complex patients: protocol for a scoping review. BMJ Open.

[16] Udeh NN, Idemili Aronu N, Ezeome ER (2026). A qualitative study of the ethical issues encountered at end-of-life care at a university teaching hospital in Nigeria. J Med Ethics.

[17] Piscitello G, Wolwowicz E, Huber M, Vranas KC, Sullivan DR, Hauschildt K, Lyons PG (2025). How clinicians approach withholding or withdrawing life-sustaining treatment in ethically controversial scenarios. Am J Respir Crit Care Med.

[18] Kim SH, Jang JH, Kim YZ, Kim KH, Nam TM (2024). Recent trends in the withdrawal of life-sustaining treatment in patients with acute cerebrovascular disease: 2017-2021. J Korean Neurosurg Soc.

[19] Martins CS, Nunes R (2024). Portuguese advance directives - a twist against futility? A cross sectional study. Sao Paulo Med J.

[20] Nieder C, Mannsåker B, Yobuta R, Haukland E (2020). Provider decision regret—a useful method for analysis of palliative thoracic re-irradiation for lung cancer?. Strahlenther Onkol.

[21] Carter HE, Lee XJ, Gallois C (2019). Factors associated with non-beneficial treatments in end of life hospital admissions: a multicentre retrospective cohort study in Australia. BMJ Open.

[22] Chamberlin P, Lambden J, Kozlov E (2019). Clinicians’ perceptions of futile or potentially inappropriate care and associations with avoidant behaviors and burnout. J Palliat Med.

[23] Kamat S, Rajendram P, Shuman A (2012). A contemporary analysis of ethics consultations in an oncologic ICU. Chest.

[24] Lopez A, Yager J, Feinstein RE (2010). Medical futility and psychiatry: palliative care and hospice care as a last resort in the treatment of refractory anorexia nervosa. Int J Eat Disord.

[25] Palda VA, Bowman KW, McLean RF, Chapman MG (2005). "Futile" care: do we provide it? Why? A semistructured, Canada-wide survey of intensive care unit doctors and nurses. J Crit Care.

[26] Rivera S, Kim D, Garone S, Morgenstern L, Mohsenifar Z (2001). Motivating factors in futile clinical interventions. Chest.

[27] Curtis JR, Patrick DL, Caldwell ES, Collier AC (2000). The attitudes of patients with advanced AIDS toward use of the medical futility rationale in decisions to forego mechanical ventilation. Arch Intern Med.

[28] Bloomer MJ, Tiruvoipati R, Tsiripillis M, Botha JA (2010). End of life management of adult patients in an Australian metropolitan intensive care unit: a retrospective observational study. Aust Crit Care.

[29] Lonergan B, Wright A, Markham R, Machin L (2020). Time-limited trials: a qualitative study exploring the role of time in decision-making on the intensive care unit. Clin Ethics.

[30] Willmott L, White B, Gallois C (2016). Reasons doctors provide futile treatment at the end of life: a qualitative study. J Med Ethics.

[31] Beck S, van de Loo A, Reiter-Theil S (2008). A "little bit illegal"? Withholding and withdrawing of mechanical ventilation in the eyes of German intensive care physicians. Med Health Care Philos.

[32] Lee SI, Hong KS, Park J, Lee YJ (2020). Decision-making regarding withdrawal of life-sustaining treatment and the role of intensivists in the intensive care unit: a single-center study. Acute Crit Care.

[33] Chauchard C, Goury A, Lafon B (2026). Withholding and withdrawal of care in the ICU of eastern France modalities and families feeling. Palliat Support Care.

[34] Lee YL, Ong YY, Thong SY, Ng SY (2018). A retrospective study of end-of-life care decisions in the critically ill in a surgical intensive care unit. Indian J Palliat Care.

[35] Druwé P, Monsieurs KG, Piers R (2018). Perception of inappropriate cardiopulmonary resuscitation by clinicians working in emergency departments and ambulance services: the REAPPROPRIATE international, multi-centre, cross sectional survey. Resuscitation.

[36] Zier LS, Burack JH, Micco G, Chipman AK, Frank JA, White DB (2009). Surrogate decision makers' responses to physicians' predictions of medical futility. Chest.

[37] Kadooka Y, Asai A, Bito S (2012). Can physicians' judgments of futility be accepted by patients? A comparative survey of Japanese physicians and laypeople. BMC Med Ethics.

[38] Pasieka PM, Skupnik W, Fronczek M, Jaeschke R, Szczeklik W (2025). The experiences and opinions of Polish medical personnel regarding limitations of futile treatment in intensive care units - a questionnaire study. Palliat Support Care.

[39] Serenari M, Berti D, Rivera B (2025). Optimizing outcomes in gallbladder cancer: identifying predictors of futile up-front surgery in a global multi-center study. Ann Surg Oncol.

[40] Hariharan S, Moseley HS, Kumar AY, Walrond ER, Jonnalagadda R (2003). Futility-of-care decisions in the treatment of moribund intensive care patients in a developing country. Can J Anaesth.

[41] Jukić M, Šarić L, Prkić I, Puljak L (2016). Medical futility treatment in intensive care units. Acta Med Acad.

[42] Cruz VM, Camalionte L, Caruso P (2015). Factors associated with futile end-of-life intensive care in a cancer hospital. Am J Hosp Palliat Care.

[43] Lo JJ, Yoon S, Neo SH, Sim DK, Graves N (2022). Factors influencing potentially futile treatments at the end of life in a multiethnic Asian cardiology setting: a qualitative study. Am J Hosp Palliat Care.

[44] Kalkan EA, Mirici A (2018). Opinions of chest physicians about the do-not-resuscitate (DNR) orders: respect for patient’s autonomy or medical futility?. J Crit Intensive Care.

[45] Aizawa K, Asai A, Bito S (2013). Defining futile life-prolonging treatments through neo-Socratic dialogue. BMC Med Ethics.

[46] Becerra-Bolaños Á, Ramos-Ahumada DF, Herrera-Rodríguez L, Valencia-Sola L, Ojeda-Betancor N, Rodríguez-Pérez A (2024). Withdrawal/withholding of life-sustaining therapies: limitation of therapeutic effort in the intensive care unit. Medicina (Kaunas).

[47] Ferrand E, Marty J (2006). Prehospital withholding and withdrawal of life-sustaining treatments. The French LATASAMU survey. Intensive Care Med.

[48] Azoulay E, Garrouste M, Goldgran-Toledano D (2012). Increased nonbeneficial care in patients spending their birthday in the ICU. Intensive Care Med.

[49] Robertsen A, Helseth E, Laake JH, Førde R (2019). Neurocritical care physicians' doubt about whether to withdraw life-sustaining treatment the first days after devastating brain injury: an interview study. Scand J Trauma Resusc Emerg Med.

[50] Huang YC, Huang SJ, Ko WJ (2010). Survey of do-not-resuscitate orders in surgical intensive care units. J Formos Med Assoc.

[51] Parrish J, Neville TH, Tarn DM, Chang DW (2026). Decision-making about potentially non-beneficial intensive care unit treatments: interviews of family members from an academic public hospital. J Intensive Care Med.

[52] Monsalve G, Echeverri J, Echeverri J, Quintero ET (2026). Clinical and ethical approaches to medical futility in end-of-life care: a scoping review. OSF.

